# Development and Validation of Creatinine-Based Estimates of the Glomerular Filtration Rate Equation from ^99m^Tc-DTPA Imaging in the Malaysian Setting

**DOI:** 10.1155/2021/3465472

**Published:** 2021-09-08

**Authors:** Azrina Md Ralib, Farah Nadia Mohd Hanafiah, Iqbalmunawwir Abd Rashid, Mohamad Shahrir Abd Rahim, Fatimah Dzaharudin, Mohd Basri Mat Nor

**Affiliations:** ^1^Department of Anaesthesiology and Intensive Care, Kulliyyah of Medicine, International Islamic University Malaysia, Kuantan, Pahang, Malaysia; ^2^Department of Radiology, Kulliyyah of Medicine, International Islamic University Malaysia, Kuantan, Pahang, Malaysia; ^3^Department of Mechanical Engineering, Kulliyyah of Engineering, International Islamic University Malaysia, Gombak, Selangor, Malaysia

## Abstract

**Introduction:**

Accurate assessment of glomerular filtration rate (GFR) is very important for diagnostic and therapeutic intervention. Clinically, GFR is estimated from plasma creatinine using equations such as Cockcroft–Gault, Modification of Diet in Renal Disease, and Chronic Kidney Disease-Epidemiology Collaboration (CKD-EPI) equations. However, these were developed in the Western population. To the best of our knowledge, there was no equation that has been developed specifically in our population.

**Objectives:**

We developed a new equation based on the gold standard of ^99m^Tc-DTPA imaging measured GFR. We then performed an internal validation by comparing the bias, precision, and accuracy of the new equation and the other equations with the gold standard of ^99m^Tc-DTPA imaging measured GFR.

**Methods:**

This was a cross-sectional study using the existing record of patients who were referred for ^99m^Tc-DTPA imaging at the Nuclear Medicine Centre, International Islamic University Malaysia. As this is a retrospective study utilising routinely collected data from the existing pool of data, the ethical committee has waived the need for informed consent.

**Results:**

Data of 187 patients were analysed from January 2016 to March 2021. Of these, 94 were randomised to the development cohort and 93 to the validation cohort. A new equation of eGFR was determined as 16.637 ∗ 0.9935^Age^ ∗ (SCr/23.473)^−0.45159^. In the validation cohort, both CKD-EPI and the new equation had the highest correlation to ^99m^Tc-DTPA with a correlation coefficient of 0.81 (*p* < 0.0001). However, the new equation had the least bias and was the most precise (mean bias of −3.58 ± 12.01) and accurate (P30 of 64.5% and P50 of 84.9%) compared to the other equations.

**Conclusion:**

The new equation which was developed specifically using our local data population was the most accurate and precise, with less bias compared to the other equations. Further study validating this equation in the perioperative and intensive care patients is needed.

## 1. Introduction

Accurate assessment of glomerular filtration rate (GFR) in perioperative and intensive care patients is very important for diagnostic and therapeutic intervention [1, 2]. Clinically, GFR is estimated from plasma creatinine using equations such as Cockcroft–Gault (CG) [3], Modification of Diet in Renal Disease (MDRD) [4], and Chronic Kidney Disease-Epidemiology Collaboration (CKD-EPI) equations [5]. However, these were developed in the Western population. These equations had different performances depending on different ethnic groups [6–8]. Specifically in other populations, coefficients has been added in the equations in order to make the GFR estimating equations more accurate such as for the South African, Chinese, Korean, Thailand, and Japanese populations [9–16].

In our local setting, three studies by the same author had been conducted comparing the established equation with ^51^chromium ethylenediamine tetra acetic acid (^51^Cr-EDTA) clearance as the gold standard [17–19]. These studies only compared established equations without addition of a racial coefficient. Another study in our neighbouring country showed that addition of an ethnic coefficient did not improve the performance of the CKD-EPI equation when compared to the gold standard of Technetium-99m-diethylenetriaminepentaacetic acid (^99m^Tc-DTPA) GFR measurement in 232 multiethnic groups of patients [20]. To the best of our knowledge, there was no previous study that has developed eGFR specifically in our population. We proposed a new equation based on the gold standard of ^99m^Tc-DTPA imaging measured GFR that can be used specifically in our population. We then performed an internal validation of the new equation compared to the other established equations in our population of patients.

## 2. Materials and Methods

This was a cross-sectional study using the existing record of patients that was referred for ^99m^Tc-DTPA imaging at the Nuclear Medicine Centre, International Islamic University Malaysia. Inclusion criteria were patients older than 16 years of age who were referred to the centre from January 2016 to March 2021. Exclusion criteria were those with missing data. Ethical approval was obtained from the IIUM Research Ethics Committee (IREC Number 2019-153). As this is a retrospective study whereby data were collected from the existing pool of data, the ethical committee waived the need for informed consent.

Patients who came were advised to be well hydrated and requested to void just prior to the procedure. 0.8–10 mCi of ^99m^Tc-DTPA was administered intravenously. Intravenous frusemide 20 mg was given 20 minutes from the start of the procedure. The renal dynamic imaging measurements were then carried out and after images acquisition, and measured GFR were automatically calculated by using the computer using the Gates method. Estimated glomerular filtration rate were calculated based on the Cockcroft–Gault, MDRD, and CKD-EPI equations. Demographics data of the patients that were collected include age, gender, height, weight, and ethnicity. The clinical data that were collected include CKD aetiology, background comorbidities, and vitals sign and laboratory data included the renal function test. The ^99m^Tc-DTPA readings were also recorded. If there were multiple measurements of scans performed, the latest ^99m^Tc-DTPA was taken. The renal function test must be within three months of when the ^99m^Tc-DTPA imaging was conducted.

Patients were randomised into a development and validation cohort using a random number generated in Microsoft Excel. In the development cohort, a new equation was developed using a nonlinear regression model utilising a generalized least squares algorithm. Internal validation was performed in the validation cohort, by comparing the bias, precision, and accuracy of the new equations and the other established equations to the measured GFR.

### 2.1. Statistical Analysis

Results were presented as mean ± SD for normally distributed variables or median (interquartile range) for nonnormally distributed variables. Comparison of variables between the two groups was analysed using the independent *t*-test for normally distributed variables or the Mann–Whitney test for nonnormally distributed variables. Categorical variables were compared with the chi-square test. Spearman or Pearson rank correlation and linear regression were used to assess the relationship between estimated and measured GFRs. Agreement between estimated and measured GFRs was tested with the Bland–Altman plot. Bias, precision, and accuracy were tested to compare for all these equations. Bias was denoted as the area between the regression line and distance along the zero difference line. The differences between estimated and measured GFRs were regressed against the average of estimated and measured GFRs. The percent difference (relative bias) was calculated as (absolute median difference/measured GFR) × 100 [1, 16]. Precision was denoted by the standard deviation of the mean difference between measured and estimated GFRs. Accuracy was calculated by the proportion of eGFR values that is within 30% of measured GFR and that within 50% measured GFR [16].

## 3. Results

**Figure 1:**
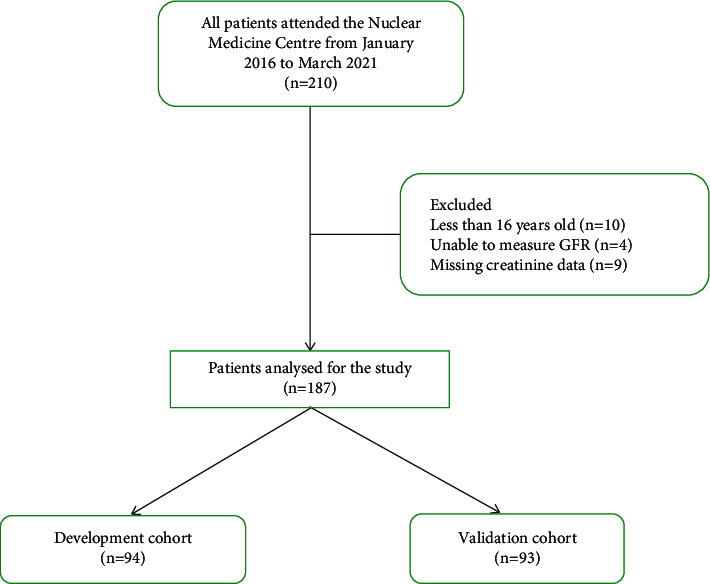
Flow chart of patients' recruitment.

A total of 210 patients attended the centre. However, 10 were excluded as they were less than 16 years of age, four with GFR not being able to be measured, and nine due to missing creatinine data (Figure 1). Data of 187 patients were analysed; of these, 94 were randomised to the development cohort and 93 to the internal validation cohort.

### 3.1. Demographic and Clinical Characteristics

Table 1 shows the demographic and clinical characteristics of patients in the development cohort. There were no differences in the demographic and clinical characteristics between patients in the development and validation cohorts. In addition, there were no differences in plasma creatinine and measured and all estimated GFRs between the two cohorts of patients.

### 3.2. Development Cohort (*n* = 94)

**Figure 2:**
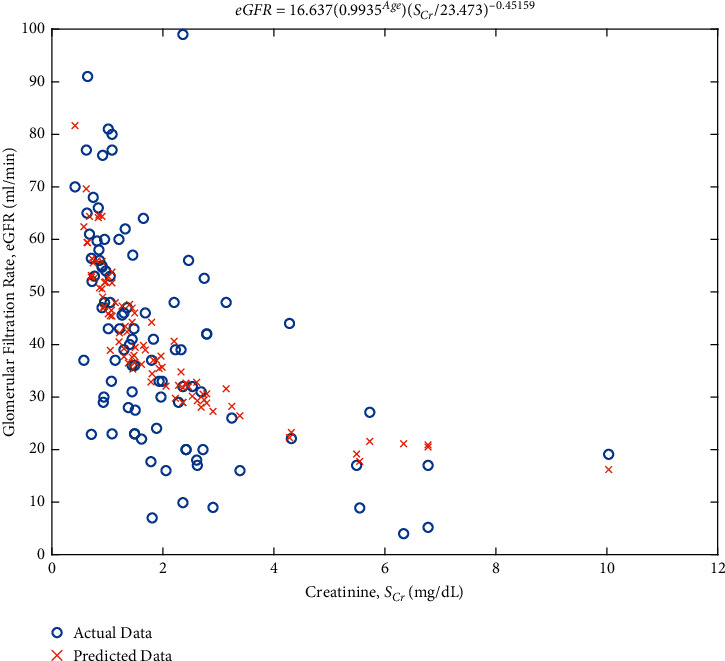
Linear egression model of glomerular filtration rate and serum creatinine for actual data (blue circle) of 94 patients in the development cohort. A new equation was developed using generalized least square algorithm with the best prediction (orange cross).

A new equation was developed in 94 patients randomised to the development cohort. The equation determined is eGFR = 16.637 ∗ 0.9935^Age^ ∗ (SCr/23.473)^−0.45159^ (Figure 2) This equation was developed using generalized least square algorithm that predicts the new equation from the actual data of 94 patients. Regression coefficient of age and gender was added, but the final equation only had age, as the equation that best fit was similar for male and female. The *R*-square of the linear regression was 0.416, with an adjusted value of 0.404 and *F* statistic of 237.

### 3.3. Internal Validation (*n* = 93)

**Figure 3:**
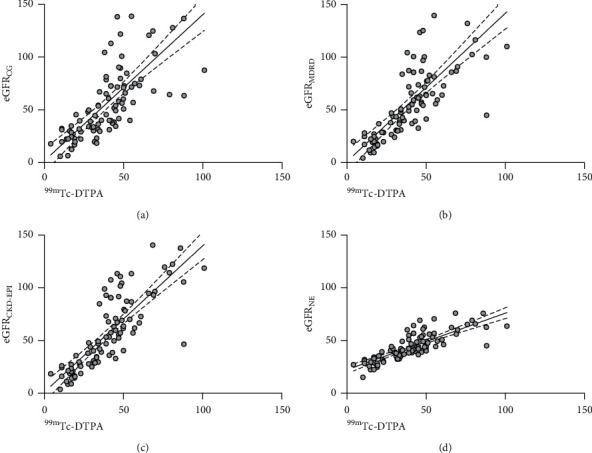
Scatter plots of eGFRs and ^99m^TC-DTPA measured GFRs for (a) eGFR_CG_, (b) eGFR_MDRD_, (c) eGFR_CKD-EPI_, and (d) eGFR_NE_. The dotted line shows the regression line and 95% confidence interval. ^99m^Tc-DTPA, Technetium-99m-diethylenetriaminepentaacetic acid; eGFR, estimated glomerular filtration rate; CG, Cockcroft–Gault; MDRD, Modification of Diet in Renal Disease; CKD-EPI, Chronic Kidney Disease-Epidemiology Collaboration; and NE: new equation.

**Figure 4:**
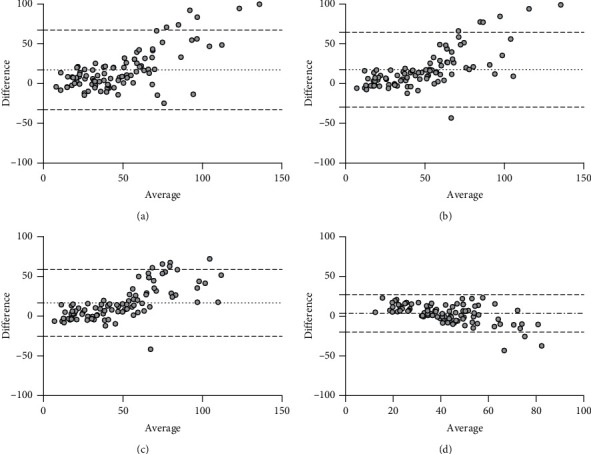
Bland–Altman plots of eGFRs and ^99m^TC-DTPA measured GFRs for (a) eGFR_CG_, (b) eGFR_MDRD_, (c) eGFR_CKD-EPI_, and (d) eGFR_NE_. The dotted line shows the mean of bias, and the bold dashed line depicts 1.96 SD of the mean bias.

An internal validation was performed in 93 patients randomised to the validation cohort. Measured GFR by ^99m^Tc-DTPA imaging was lower compared to all estimated values by the equations studied (Table 1). Both eGFR_NE_ and eGFR_CKD-EPI_ had the highest correlation of 0.81 (*p* < 0.0001) compared to the other equations (Table 2). In addition, both had the highest *R*-square of 0.66 (Table 2 and Figure 3). Linear regression analyses showed that eGFR_NE_ had the lowest coefficient constant (Table 3). The value of measured GFR can be calculated as equal to 1.22 (eGFR_NE_)–13.22 ml/min. Bland–Altman analyses of the comparison showed that eGFR_NE_ had the least bias of 3.58 ml/min compared to eGFR_CG_, eGFR_MDRD_, and eGFR_CKD-EPI_. (Table 4 and Figure 4). In addition, eGFR_NE_ also was the most precise as depicted with its lowest standard deviation of bias of 12.01 ml/min compared to the other equations. Compared to the other equations, eGFR_NE_ had the highest P_30_ and P_50_ of 64.5 and 84.9%, respectively.

## 4. Discussion

Our study aimed to develop a new equation based on the gold standard of ^99m^Tc-DTPA imaging measured GFR. From the development cohort of 94 patients, a new equation was developed using the generalized least squares algorithm. The internal validation of 93 patients showed that both CKD-EPI and the new equation had the highest correlation to the ^99m^Tc-DTPA measured GFR; however, the new equation had the least bias and was the most precise compared to the other equations.

Limitations associated with the development of the Cockcroft–Gault, MDRD, and CKD equations further preclude their use in our settings. The Cockcroft–Gault equation developed in 1976 was derived from 249 patients by using creatinine clearance, and as creatinine clearance can overestimate GFR, Cockcroft–Gault can also overestimate GFR [3]. The equation was developed in predominantly white males and did not take into account of ethnicity. The MDRD equation was derived from 1628 patients with CKD by using renal clearance of ^125^I-iothalamate [4]. The CKD-EPI equation was developed to improve the estimation of GFR by MDRD in patients with normal renal function. It involved 10 studies comprising 8254 participants [5]. Ethnicity factor in both MDRD and CKD-EPI is confined to the Western populations comprising black (African-American) and nonblack, based on the assumption that the black ethnic group has higher muscle mass.

Ethnicity plays a role in estimating the glomerular filtration rate [6, 8]. Several studies conducted in Asian populations in the past several years have proposed a revised formula or addition of a racial coefficient to improve the estimates of GFR in the Chinese [13–15], Japanese [7, 9], South African [16], Korean [10], and Thailand [11] populations. Most of the studies added a racial coefficient to the existing equation which was shown to be more accurate than the original equation [9, 21]. One study developed a revised CKD-EPI equation in 960 Korean patients against ^51^Cr-EDTA clearance; however, it was shown to be equivalent to the original CKD-EPI equation [10]. Nevertheless, most of the studies recommended that each population should develop and validate eGFR equations specific to the population prior to the epidemiologic and clinical use of the equations.

To the best of our knowledge, there is no study conducted that develops an equation or coefficient in our local setting. Comparative studies have been conducted by various investigators in investigating the utility of the established equations to the gold standard of measured GFR. However, this is the first study that developed the eGFR equation in our setting which comprises predominantly the Malay population. In our local setting, two studies had compared the established equation to the gold standard of ^51^Cr-EDTA clearance. MDRD was shown to be the most accurate when compared to the other CKD-EPI and Cockcroft–Gault equations in 51 elderly Malay patients [17]. In analysis of 113 multiethnic groups of patients, CKD-EPI was shown to be the most precise and accurate compared to MDRD.

Creatinine remains the only available biomarker to evaluate kidney function in our local setting, and hence, the creatinine-based formula remained an important tool for assessment of kidney function. Cystatin C is a newer functional marker to assess kidney function; however, it is not widely available. Nevertheless, Jalalonmuhali compared creatinine-based equations to the cystatin C-based equation, of which creatinine-based equation still performed better in 40 elderly patients [19]. One study compared the original equation and those that were racially adjusted of 232 multiethnic patients in Singapore (94 Chinese, 74 Malay, and 64 Indians and others). Comparing these to the ^99m^Tc-DTPA GFR measurement, the author showed that the original CKD-EPI equation performed better than that was racially adjusted [20].

The gold standard of GFR measurement is clearance of an ideal filtration marker, that is, one which is freely filtered in the glomerulus and is neither secreted, reabsorbed, synthesised, nor metabolised by the kidney. Gold-standard markers used include inulin and radioactive markers, such as ^51^Cr-EDTA [12, 22] or ^99m^Tc-DPTA [23]. However, in most cases, they are expensive, difficult to assay, and require a continuous infusion. Hence, their use is usually limited to research studies. Hence, development of a new equation, specifically in our setting utilising the gold standard, could overcome the impediment. Our study utilised the gold standard of GFR measurement from the ^99m^Tc-DTPA imaging conducted in our nuclear medicine clinic. Limitation of ^99m^Tc-DTPA includes that it is subjected to tubular reabsorption, protein binding, and extrarenal clearance that can vary between individuals [24]. The actual GFR can be underestimated by ^99m^Tc-DTPA renal dynamic imaging because of a very small portion of ^99m^Tc-DTPA that is bound to plasma proteins, but this is still theoretically speculated, not dependent on pathological biopsy, and is typically ignored [12]. Our finding showed that measured GFR by ^99m^Tc-DTPA imaging was lower compared to all the estimated values by all the equations.

### 4.1. Limitations of the Study

This study has several limitations. First, this study was conducted in a single centre whereby all of the patients were referred to the nuclear medicine clinic; hence, this does not reflect the general population in Malaysia. Second, as this was a retrospective study, we could only use the available recorded data and the comorbidities were not documented properly. Third, since the study was conducted in the East Coast of Malaysia where the majority of the population is Malay, we were unable to validate in the different ethnicities in the Malaysian population. Fourth, the sample size was small for a robust equation to be developed. Nevertheless, this could be an impetus for a larger study involving several centres that could further develop or validate the equation using the same method in our setting.

## 5. Conclusions

The new equation which was developed specifically using our local data population was the most accurate and precise, with less bias compared to the other equations. Further study validating this equation in the perioperative and intensive care patients is needed.

## Figures and Tables

**Table 1:** Demographic and clinical characteristics.

Variables	All patients (*n* = 187)	Development cohort (*n* = 94)	Validation cohort (*n* = 93)	*p* value
Age (years)	55.3 ± 14.2	55.9 ± 14.1	54.7 ± 14.3	0.56
Gender (male)	97 (51.9)	46 (48.9)	51 (54.8)	0.41
Weight (kg)	65 ± 14	65 ± 15	65 ± 13	0.52
Height (cm)	158 ± 10	159 ± 10	158 ± 10	0.76
Body mass index (kg/m^2^)	26.0 ± 5.4	25.9 ± 5.4	26.1 ± 5.4	0.54
Ethnicity				0.36
(i) Malay	1165 (88.2)	84 (89.4)	81 (87.1)	
(ii) Chinese	16 (8.6)	9 (9.6)	7 (7.5)	
(iii) Indian	2 (1.1)	0 (0)	2 (2.2)	
(iv) Orang Asli	2 (1.1)	1 (1.1)	1 (1.1)	
(v) Others	2 (1.1)	0 (0)	2 (2.2)	
^99m^Tc-DTPA measured GFR (ml/min)	40.6 ± 19.9	40.7 ± 20.1	40.4 ± 19.8	0.58
Plasma creatinine (*μ*mol/l)	124 (86–209)	132 (86–214)	117 (87–208)	0.33
Estimated GFR by Cockcroft–Gault (eGFR_CG_) (ml/min)	46.2 (28.5–72.6)	45.2 (26.8–71.1)	49.2 (31.1–72.8)	0.34
Estimated GFR by MDRD (eGFR_MDRD_) (ml/min)	49.4 (27.1–73.0)	46.4 (24.9–66.7)	50.8 (28.7–82.1)	0.25
Estimated GFR by CKD-EPI (eGFR_CKD-EPI_) (ml/min)	47.8 (26.0–74.7)	46.8 (23.7–69.0)	49.7 (28.2–85.8)	0.24
Estimated GFR by the new equation (eGFR_NE_) (ml/min)	43.0 ± 13.2	42.0 ± 13.2	43.9 ± 13.1	0.91

Data are expressed as mean ± SD, *n* (%), or median (lower quartile–upper quartile). BMI, body mass index; ^99m^Tc-DTPA, Technetium-99m-diethylenetriaminepentaacetic acid, eGFR, estimated glomerular filtration; MDRD, Modification of Diet in Renal Disease; CKD-EPI, Chronic Kidney Disease-Epidemiology Collaboration; NE, new equation.

**Table 2:** Correlation analyses between eGFR and ^99m^Tc-DTPA.

Variables		*r*	95% confidence interval	*R*-square	*p*
^99m^Tc-DTPA measured GFR	eGFR_CG_	0.75	0.64 to 0.83	0.56	*p* < 0.0001
	eGFR_MDRD_	0.78	0.68 to 0.85	0.60	*p* < 0.0001
	eGFR_CKD-EPI_	0.81	0.72 to 0.87	0.66	*p* < 0.0001
	eGFR_NE_	0.81	0.72 to 0.87	0.66	*p* < 0.0001

^99m^Tc-DTPA, Technetium-99m-diethylenetriaminepentaacetic acid; eGFR, estimated glomerular filtration rate; CG, Cockcroft–Gault; MDRD, Modification of Diet in Renal Disease; CKD-EPI, Chronic Kidney Disease-Epidemiology Collaboration; NE: new equation.

**Table 3:** Linear regression eGFR and ^99m^Tc-DTPA measured GFR.

Dependent variable	Independent variable	Coefficient	*p* value
^99m^Tc-DTPA measured GFR	eGFR_CG_	0.40	*p* < 0.0001
	Constant	17.14	
	eGFR_MDRD_	0.43	*p* < 0.0001
	Constant	15.61	
	eGFR_CKD-EPI_	0.47	*p* < 0.0001
	Constant	13.48	
	eGFR_NE_	1.22	*p* < 0.0001
	Constant	-13.22	

^99m^Tc-DTPA, Technetium-99m-diethylenetriaminepentaacetic acid; eGFR, estimated glomerular filtration rate; CG, Cockcroft–Gault; MDRD, Modification of Diet in Renal Disease; CKD-EPI, Chronic Kidney Disease-Epidemiology Collaboration; NE: new equation.

**Table 4:** Bland–Altman analyses between eGFR and ^99m^Tc-DTPA.

	Mean bias (ml/min)	Standard deviation of bias (ml/min)	Differences	Percent differences	P30%	P50%
eGFR_CG_	17.19	25.55	10.27 (−0.76–25.9)	34.72 (−2.07–77.14)	35.5	50.5
eGFR_MDRD_	17.42	24.03	12.04 (3.21–24.5)	30.14 (7.88–61.23)	28.0	45.2
eGFR_CKD-EPI_	16.64	21.40	12.27 (2.03–27.24)	34.91 (6.53–58.70)	26.9	46.2
eGFR_NE_	3.58	12.01	5.4 (−3.06–12.94)	14.11 (−6.33–14.11)	64.5	84.9

^99m^Tc-DTPA, Technetium-99m-diethylenetriaminepentaacetic acid; eGFR, estimated glomerular filtration rate; CG, Cockcroft–Gault; MDRD, Modification of Diet in Renal Disease; CKD-EPI, Chronic Kidney Disease-Epidemiology Collaboration; NE: new equation.

## Data Availability

Data are available on request to the corresponding author.
